# Association Between Acute Otitis Media and Inner Ear Disorders Among Adults in Aseer Region

**DOI:** 10.7759/cureus.19556

**Published:** 2021-11-14

**Authors:** Ali Maeed Al- Shehri, Ahmed S AL-Zomia, Ahmed F Alayash, Aljohrah M. Al Hunaif, Abdulrhman A. Mansour, Mushary Alqahtani, Omar A. Asiri, Saeed A. Alserhan

**Affiliations:** 1 ENT, College of Medicine, King Khalid University (KKU), Abha, SAU; 2 Surgery, College of Medicine, King Khalid University (KKU), Abha, SAU; 3 Medicine, College of Medicine, King Khalid University (KKU), Abha, SAU

**Keywords:** treatment, symptoms, pain, diseases, otitis media

## Abstract

Introduction

Acute otitis media (AOM) is an infection of the middle ear that produces pain, fever, and discharge, as well as hearing loss. It is one of the most common problems that pediatricians encounter. Almost 80% of children have had at least one episode of AOM, and between 80% and 90% have had at least one episode of otitis media with effusion before entering school.

Methods

The cross-sectional study is conducted among male and female patients, adults, and children who visited two of the largest government hospitals in the Aseer region in Southern Saudi Arabia (Aseer Central Hospital and Khamis Mushait General Hospital). The children and adults with AOM who visited the hospitals were traced by searching the medical record system by the keyword “acute otitis media.” Two authors extracted data from the medical record and patients. After extracting data, the patient will be called through mobile phone to invite them to participate in the study. If the patient agrees to participate, she/he would be sent through email link containing an encrypted and high-security electronic signature to obtain his/her consent.

Conclusion

One of the most common pediatric infections is otitis media (inflammation of the middle ear). Children are more often than adults to get otitis media, and the majority of cases are treated with antibiotics. Clinicians commonly miss the acute stage of the disease, especially in children under the age of five. Delay or omission of diagnoses leads to inefficient management and an increased risk of negative effects.

## Introduction

Acute otitis media (AOM) is a middle ear infection that causes discomfort, fever, and occasional discharge, as well as hearing loss (Zakzouk et al., 2002) [1]. Around 80% of children have at least one episode of AOM [2]. In one study, 80-90% of the subjects had at least one episode of otitis media [3]. One study stated that acute suppurative otitis media (ASOM) usually causes severe deep ear pain [4]. As per research, purulence in the middle ear is also present in the mastoid air cells because they are connected [5,6].

According to one study, AOM may be complicated by an inner ear disorder (IED), which damages the basal cochlear turn (localized serous or toxic labyrinthitis) [6]. A Korean study discovered an incidence of 9.6% in 75 patients with AOM, with sensorineural hearing loss (SNHL) occurring within zero to 10 days of AOM onset and regularly beginning with high-frequency hearing loss [7]. In Saudi Arabia, the number of incidences of AOM in children varies in different provinces, with the southern and central provinces having the highest frequency [8]. The incidence was more significant in young children under four years and decreased in children aged eight to 12 years [9]. Male children had a slightly higher rate of AOM than female children (1.36% vs. 0.80%) [10].

According to one study, there are several universally accepted risk factors for AOM, including allergies, craniofacial deformities, iron deficiency, passive smoking, and hypertrophic adenoids [11]. Mastoiditis, a severe bacterial infection affecting the mastoid bone behind the ear, is the most prevalent complication currently. Infection of the inner ear is most typically observed in people who do not have otitis media [12]. It is frequently self-limited and linked with a nonspecific viral disease [13]. When it comes to otitis media, though it's usually a bacterial infection that has to be treated quickly.

## Materials and methods

The cross-sectional study was conducted among patients - male and female, adults and children - who visited two of the largest government hospitals in the Aseer region in Southern Saudi Arabia (Aseer Central Hospital and Khamis Mushait General Hospital). The children and adults with AOM who visited the hospitals were traced by searching the medical record system using the "acute otitis media." Two authors extracted data from the medical record and patients. After extracting the data, the patient will be called by mobile phone to invite them to participate in the study. If the patient agrees to participate, she/he would be sent an email link containing an encrypted and high-security electronic signature to obtain his/her consent. After the consent is electronically signed, the patient will be asked about presenting symptoms of AOM, and all relevant clinical data, which will be stored in an encrypted electronic data capture system (Castor EDC®). If the patient refuses to participate, we will not collect his or her data.

Regarding the questionnaire, it was constructed by a panel of experts after the discussions. Statistical analysis was performed using SPSS version 26.0 software (Armonk, NY: IBM Corp.). Categorical variables are presented using descriptive statistics, including total numbers and percentages. Comparisons between categorical variables are analyzed using a chi-square test. Continuous variables are presented as means with standard deviation (SD). A p-value < 0.05 is considered statistically significant. The sampling technique was a convenient method of sampling, with those who had visited hospitals with ear diseases during the study period being included in this study. The study was approved by the general directors of the health affairs-Aseer region KSA. IRB approval number REC-No (14-07-2021).

## Results

Out of 229 respondents, 37.1% were females while 62.9% were males; 48.8% had a university-level education, 63.3% were married, and 36.7% were single; 46.3% had diseases related to the nose, 93.4% were Saudi and 6.6% were non-Saudi; 21.8% were unemployed, and 89.5% were from the Aseer region (Table 1). In our study, we observed that 34.9% have inner ear diseases, 59.8% have pain in the ear, and 52.0% have suffered from complications after the middle ear infection. The majority of the respondents (81.2%) had a disability due to otitis media for less than six months. In our research, 48.0% of the infection period was last year (Tables 2-5). 

**Table 1:** Demographic variables

Demographic variables	Frequency	%
Do you suffer from any disease in the nose?		
No	123	53.7
Yes Yes	106	46.3
Nationality		
Non -Saudi	15	6.6
Saudi	214	93.4
Occupation		
Army	24	10.5
Bachelor's degree in Information Systems	1	0.4
Doctor	10	4.4
Engineer	5	2.2
Freelancing	1	0.4
Government employee	34	14.8
Health educator	1	0.4
Housewife	6	2.6
Nurse	1	0.4
Public sector	2	0.9
Private Sector	8	3.5
Retired	10	4.4
Student	45	19.7
Teacher	29	12.7
Unemployed	50	21.8
Worker	2	0.9
Residence		
Aseer Region	205	89.5
Outside the Aseer region	24	10.5
Total	229	100.0
Marital status		
Married	145	63.3
Single	84	36.7
Age (years)		
>60	16	7.0
19-29	51	22.3
30-39	67	29.3
40-49	42	18.3
50-59	24	10.5
6-18	29	12.7
Education		
Diploma	2	0.9
Elementary or lower	44	19.2
Middle	22	9.6
Postgraduate	20	8.7
Secondary	43	18.8
University	98	42.8
Gender		
Female	85	37.1
Male	144	62.9

**Table 2:** Items related to pain

Items related to pain	Frequency	%
Do you suffer from diseases of the inner ear?		
No	149	65.1
Yes	80	34.9
Is there pain in the ear?		
No	92	40.2
Yes	137	59.8
Have you suffered from complications after the infection of the middle ear?		
No	110	48.0
Yes	119	52.0

**Table 3:** Diagnostic period of otitis media

How long have you been diagnosed with otitis media?	Frequency	%
14 years	1	0.4
20 years	1	0.4
30 years	1	0.4
6 months to 1 year	29	12.7
6 years	3	1.3
7 years	1	0.4
8 years	1	0.4
Less than 6 months	186	81.2
No	6	2.6
Total	229	100.0

**Table 4:** Types of otitis media (OTM)

What type of otitis media do you have?	Frequency	%
Acute non-purulent otitis media	21	9.2
Acute suppurative otitis media	29	12.7
Otitis media with no effusion	27	11.8
Otitis media, unspecified	152	66.4
Total	229	100.0

**Table 5:** Symptoms of acute otitis media

What symptoms have you been complaining about? (more than one answer can be chosen)	Frequency	%
Difficulty hearing or not responding to sounds	28	12.2
Difficulty hearing or not responding to sounds loss of balance, headache	2	0.9
Ear pain, but when lying down, difficulty sleeping, irritability, fever of 38°C or more, fluid draining from the ear	1	0.4
Ear pain, especially when lying down, difficulty sleeping, difficulty hearing or not responding to sounds fluid discharge from the ear	2	0.9
Ear pain, especially when lying down, difficulty sleeping, difficulty hearing or not responding to sounds loss of balance, headache, pain in joints and bones	134	58.5
Ear pain, especially when lying down, difficulty sleeping, fever of 38°C or more, fluid drainage from the ear	1	0.4
Loss of balance, headache	2	0.9
No Symptoms	59	25.8

We have observed that 59.8% were using cotton sticks for cleaning (Table 6); 34.1% were using antibiotics while 1.2% were using multiple treatments. We have noticed that 66.4% have unspecified otitis media, 11.8% have operation theater (OT) with no effusion, 12.7% have acute superlative OT, and 9.2% have acute non-purulent OT (Tables 7, 8). As per the data in Table 8, we did not observe any significant differences while comparing resident status with nose diseases. Significant differences between types of OT and inner ear diseases were observed.

**Table 6:** Treatments that have been given for the infection

What treatment have you been given for this infection? (more than one answer can be chosen)	Frequency	%
Vitamin B12 drops, all of the above	1	0.4
Antibiotics	78	34.1
Antibiotics, all of the above	2	0.9
Antibiotics, aspiration of fluids through the ear canal	8	3.5
Antibiotics, drops	1	0.4
Antibiotics, I didn't take any medicine	1	0.4
Antibiotics, pain reliever, aspiration of fluid through the ear canal	16	7.0
Antibiotics, pain reliever, aspiration of fluid through the ear canal, all of the above	5	2.2
Antibiotics, pain reliever, aspiration of fluid through the ear canal, all of the above, B12 vitamin	1	0.4
Aspiration of fluid through the ear canal	5	2.2
Drops	3	1.3
I didn't take any medicine	20	8.7
I didn't take any nougat	3	1.3
No treatment	31	13.5
No, aspiration of fluid through the ear canal	2	0.9
Operation	1	0.4
Panadol	1	0.4
Surgery	5	2.2
Surgery	1	0.4
Multiple treatments	44	19.2

**Table 7:** Comparison between nose disease with resident status

Do you suffer from any disease in the nose?		No		Yes	Total	p-Value
Residence	Aseer region	108	97		205	0.361
	Outside the Aseer region	15	9		24	
Total		123	106		229	

**Table 8:** Comparisons between type of OT media and inner ear diseases OT: operation theater

What type of otitis media do you have?	Do you suffer from diseases of the inner ear?		Total	p-Value
	No	Yes		
Acute non-purulent otitis media	13	8	21	0.018
Acute suppurative otitis media	17	12	29	
Otitis media with no effusion	11	16	27	
Otitis media, unspecified	108	44	152	

## Discussion

For this study, 229 patients were recruited from two public hospitals between March 2019 and February 2020 for the purpose to assess the incidences of various forms of AOM. Surprisingly, the occurrence of IED in AOM has only been recorded in animal studies, histological and clinical case reports, and case series, while epidemiological data on the incidence of IED in AOM is sparse [14,15]. In fact, the authors found only one study that determined the incidence of IED in AOM in a clinical setting, which retrospectively analyzed the cases of 75 adult patients (83 ears) with AOM from a secondary referral hospital and found an SNHL in 9.6% of cases (eight ears), using rather strict SNHL inclusion criteria (bone conduction {BC} loss of 30 dB in three frequencies in comparison to the opposite normal ear) [14]. 

In our study, we found that patients were taking antibiotics, which is consistent with other studies' findings. According to a meta-analysis of randomized studies, antibiotics are most beneficial in children under the age of two years who have bilateral acute otitis media, but not in children who have acute otitis media with otorrhea [16]. Antibiotics are advised for all infants under the age of six months, children aged six months to two years when the diagnosis is certain, and children aged two years and older who have a serious infection (defined as moderate to severe otalgia or a temperature of more than 102.2°F) [15]. According to our research, earache, ear discharge, hearing loss, ear-popping, ear fullness, dizziness, and fever are all indications of otitis media.

## Conclusions

Otitis media (inflammation of the middle ear) is one of the most common pediatric infections. Children are more susceptible to otitis media than adults, while most cases are treated with antibiotics. Clinicians often miss the acute phase of the disease. Diagnoses that are delayed or overlooked result in ineffective management and an increased risk of consequences. Parents will take care of their kids and give full and immediate attention to avoid further complications. The use of antibiotics is also helpful, however, only after the recommendations of the concerned doctor. Future studies are also required to explore further and educate the family to fulfill their responsibilities more appropriately.
